# Structural Features of an OR37 Glomerulus: A Comparative Study

**DOI:** 10.3389/fnana.2017.00125

**Published:** 2017-12-18

**Authors:** Anna-Maria Maier, Heinz Breer, Jörg Strotmann

**Affiliations:** Institute of Physiology, University of Hohenheim, Stuttgart, Germany

**Keywords:** olfactory bulb, periglomerular cells, OR37, glomeruli, calretinin, calbindin, parvalbumin

## Abstract

In the olfactory bulb (OB) a sophisticated neuronal network mediates the primary processing of sensory information and extensive investigations over the past decades have greatly improved our understanding of the morphology and neuronal organization of the OB. However, efforts have mostly been focused on the different radial layers, typical for the OB and little attention has been paid to individual odorant receptor specific glomeruli, the first relay station of sensory information. It has been assumed that glomeruli processing odorant information out of different contextual fields might require accordingly specialized neuronal networks. In this study, we have analyzed and compared the structural features as well as cell types in the periglomerular (PG) region of three odorant receptor specific glomeruli. The investigations were focused on glomeruli of the receptor type OR37A, a member of the unique OR37 subsystem, in comparison to glomeruli of OR18-2, a class I odorant receptor and OR256-17, a class II receptor. Each of the odorant receptor types is known to be activated by distinct odorants and their glomeruli are located in different regions of the bulb. We found significant differences in the size of the glomeruli as well as in the variability of the glomerulus size in individual mice, whereby the OR37A glomeruli featured a remarkably stable size. The number of cells surrounding a given glomerulus correlated strongly with its size which allowed comparative analyses of the surrounding cell types for individual glomeruli. The proportion of PG cells labeled by NeuN as well as putative GABAergic neurons labeled by GAD65 was quite similar for the different glomerulus types. However, the number of cells expressing distinct calcium-binding proteins, namely parvalbumin (PV), calbindin (CB) or calretinin (CR) varied significantly among the three glomerulus types. These data suggest that each odorant receptor specific glomerulus type may be surrounded by a unique network of PG cells.

## Introduction

In the past decades, great efforts have been made to explore the morphology and neuronal organization of the olfactory bulb (OB) to provide a structural basis for further insights in functional mechanisms underlying the processing of olfactory information (for review see Nagayama et al., 2014; Kosaka and Kosaka, 2016). A variety of neuronal subtypes in the OB have been studied regarding their morphology, physiology and neurochemical properties (Parrish-Aungst et al., 2007, 2011; Panzanelli et al., 2007; Bywalez et al., 2017; Pignatelli and Belluzzi, 2017). However, in most of the studies, little attention has been paid to the topographic location of the neurons along the anteroposterior or dorsoventral axes of the OB. Any distinctions were mostly based on the different radial layers of the bulb. Although, the OB is a highly heterogeneous brain region composed of several functional areas specialized in processing odor information which may be relevant for various behavioral aspects, such as foraging or mating (Auffarth et al., 2011; Mori and Sakano, 2011; Inokuchi et al., 2017). Moreover, the OB is divided in distinct zones representing the phylogenetic class of the odorant receptors (ORs) processed there, namely class I (fish-like) and class II (terrestrial-like) odorant receptors (Zhang and Firestein, 2002; Matsuo et al., 2015). The two classes are represented in a spatially segregated manner in the olfactory epithelium and also in the OB, in a ventral and a dorsal domain (Tsuboi et al., 2006; Kobayakawa et al., 2007). In accordance with the genetic and spatial classification, different functional implications of the associated glomeruli have been demonstrated in several studies (Lin et al., 2005; Kobayakawa et al., 2007; Cho et al., 2011; Inokuchi et al., 2017). For example, the processing of predator odors, aversive spoiled food odors and attractive social odors could be clearly allocated to different domains of the bulb (Kobayakawa et al., 2007; Inokuchi et al., 2017). A striking example for the heterogeneity within the olfactory system is the OR37 subsystem. Despite being part of the main olfactory system, it has a variety of unique features. Odorant receptors of the OR37 family are exclusively found in mammals and are highly conserved, even across species borders, which is highly unusual for odorant receptors (Hoppe et al., 2003, 2006). Additionally, olfactory sensory neurons which express a receptor of the OR37 subfamily are segregated in a small patch within the olfactory epithelium, neglecting the typical zonal distribution patterns (Strotmann et al., 1999). Their projecting axons form only one glomerulus in the anterior ventromedial part of the OB (Strotmann et al., 2000), a region of the bulb that is supposed to be involved in processing social odor information (Schaefer et al., 2001; Lin et al., 2007). In fact, in a previous study we have demonstrated that the exposure of mice to ligands of the OR37 subsystem mimics certain social buffering effects (Klein et al., 2015). Collectively, these data support the idea of functionally specialized regions of the OB tuned to the processing of odorants from distinct contexts, i.e., social, food or predator odors. Odorants out of different contextual fields, eliciting distinct behaviors as a consequence, might require specialized neuronal computations within certain glomeruli. Thus, it might be inappropriate to assume a uniform constitution of the interneuronal network throughout bulbar domains and distinct glomeruli. Especially for an extraordinary subsystem as the OR37, which stands out to the canonical odorant receptor classes and is probably involved in processing socially relevant odors, a distinct neuronal processing pattern seems conceivable. Therefore, in this study we aimed to characterize structural features of distinct odorant receptor specific glomeruli and in particular their periglomerular (PG) neuron subpopulations. PG cells are the most abundant interneuron type in the glomerular layer. The dendrites of one subpopulation of PG cells (type I) form direct synaptic contacts to the incoming axons of olfactory sensory neurons; these cells have therefore been categorized as olfactory nerve driven. Type II cells, in contrast, have synaptic contacts to other OB neurons, like ET cells and are likely driven by polysynaptic inputs (Kosaka and Kosaka, 2007; Shao et al., 2009). These dissimilarities in synaptic organization are thought to be responsible for the observed differences in response to olfactory nerve input (Shao et al., 2009).

Our analyses were focused on glomeruli of OR37A as a representative of the OR37 subsystem, in contrast to glomeruli of each, class I and class II odorant receptors, namely OR18-2 and OR256-17. The glomeruli of these three odorant receptor types are located in different regions of the OB and convey olfactory information out of diverging contextual fields. OR18-2 could be shown to respond to metabolic products such as acetate or propionate (Pluznick et al., 2013; Fleischer et al., 2015), whereas OR256-17 is broadly tuned to environmental cues, i.e., acetophenone or carvones (Tazir et al., 2016). The experimental studies were performed using mouse models in which glomeruli of the respective odorant receptors are transgenically labeled, allowing a specific examination of individual glomeruli.

## Materials and Methods

### Mice

Adult mice of both sexes were used in the present study, carrying an inserted reporter allele at the locus of OR37A, OR18-2 or OR256-17, respectively, to visualize corresponding glomeruli. In alternative designations, OR37A might be found as Olfr155, OR18-2 as Olfr78 or MOL2.3 and OR256-17 as Olfr15, respectively. OR37A reporter mice possess an additional IRES-tau-lacZ or, respectively, an IRES-tau-GFP allele at the OR37A locus and those for OR256-17 an IRES-tau-GFP sequence at the locus of OR256-17 (Strotmann et al., 2000; Luxenhofer et al., 2008). These strains were bred on a C57BL/6J background. Reporter mice for OR18-2 possess an IRES-GFP-IRES-tau-lacZ sequence at the OR18-2 locus and were bred from a CD-1 background (Conzelmann et al., 2000). For analyses of GAD65 expressing interneurons, the named reporter mouse strains were crossed with a transgenic mouse strain carrying a GAD65-IRES-tdTomato allele on a C57BL/6J background (Besser et al., 2015). Mice were housed in groups at a 12-h light/dark cycle at the Central Unit for Animal Research at the University of Hohenheim and had access to food and water *ad libitum*. Experiments were carried out in accordance with the Council Directive 2010/63EU of the European Parliament and the Council of 22 September 2010 on the protection of animals used for scientific purposes. The work was approved by the University of Hohenheim Animal Welfare Officer (T126/14 Phy).

### Tissue Preparation

Mice were sacrificed via cervical dislocation and subsequent decapitation. Brains and attached nasal turbinates were prepared and fixed in 4% Paraformaldehyde at 4°C for 24 h and cryoprotected by immersion in 25% sucrose in phosphate buffered saline at 4°C overnight. Subsequently, brains were embedded in tissue freezing medium on dry ice and kept at −20°C until cryosectioning. Coronal cryosections of 12 μm thickness were used for immunohistochemistry analyses.

### Immunhistochemistry

For immunhistochemistry experiments, tissue sections were washed three times in TRIS buffered saline (TBS), blocked for 30 min with 1% BSA and 0.3% Triton in TBS and incubated with the respective primary antibody (see Table 1) in the same blocking solution overnight at 4°C. For the mOR37A-lacZ reporter strain, an antibody against β-Galactosidase was applied simultaneously to visualize OR specific lacZ expression. After primary antibody incubation, sections were washed again three times in TBS and blocked for 15 min before incubating with the corresponding secondary antibody, using the same blocking solution. All secondary antibodies were used at a concentration of 1:500, except for the β-Galactosidase staining, where the secondary antibody was used at 1:2000. A list of all antibodies used is given in Table 1. Finally, sections were washed again three times before DAPI staining (dilution 1:1000 in TBS) was conducted and stopped in bidest. Control experiments were performed by omitting the primary antibody. Slides were mounted using Mowiol.

**Table 1:** Antibodies.

Primary antibodies	Code	Supplier	Dilution	Host
Calbindin	sc-7691	Santa Cruz	1:300	goat
Calretinin	sc-11644	Santa Cruz	1:300	goat
NeuN	ab177487	abcam	1:1000	rabbit
Parvalbumin	PV 25	swant	1:300	rabbit
Tyrosine Hydroxylase	ab112	abcam	1:700	rabbit
β-Galactosidase	Z3781	Promega	1:1000	mouse
**Secondary antibodies**				
Anti-rabbit Alexa Fluor 568	ab175694	abcam	1:500	donkey
Anti-goat Alexa Fluor 568	A-11057	Invitrogen	1:500	donkey
Anti-mouse Alexa Fluor 488	A-11017	Invitrogen	1:2000	goat

*List of antibodies used in immunhistochemistry experiments*.

### Image Analyses

Fluorescence multi channel images were captured using a Zeiss Axioskop 2 plus with an AxioCam MRm camera and processed with ZEN 2009 and Axiovision SE64 4.9 software (Zeiss). For tyrosine hydroxylase (TH) immunostaining experiments, additional images were taken using a Zeiss LSM 510 meta laser scanning microscope, shown in Figure 3B. Countings were performed on exported Axiovision images using ImageJ software. The region of interest around a glomerulus was outlined in multi channel images, with a maximum range of four rows of DAPI stained nuclei. Labeled cells were then counted in the ROI using the ITCN tool for nuclear stainings, except for TH staining which was counted manually as ITCN could not reliably identify cytoplasmatic staining. Counted values of each section were summed up for individual glomeruli.

**Figure 1:**
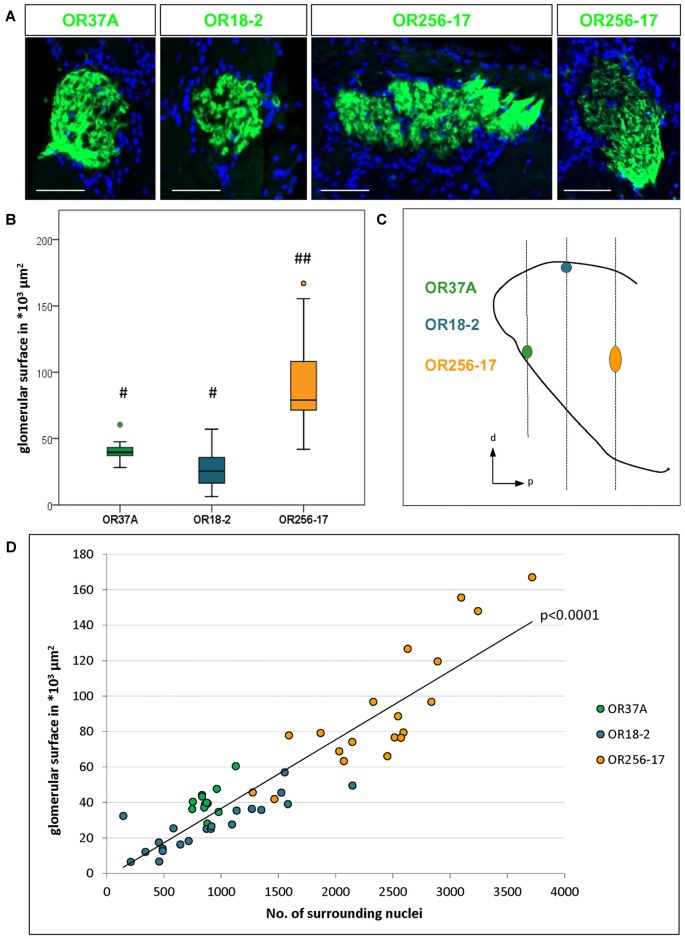
Number of surrounding cells refers to varying glomerulus size. **(A)** Glomeruli of OR37A-IRES-lacZ-tauGFP, OR18-2-IRES-lacZ-tauGFP or OR256-17-IRES-tauGFP (green) at typical sizes with surrounding DAPI stained nuclei. For OR256-17, a relatively small and a large glomerulus are shown to depict the range of variation. Scale bar: 50 μm. **(B)** Boxplot depiction of the glomerular surface in μm^2^. Glomeruli of OR37A display a medium size with remarkably little variance, whereas OR18-2 glomeruli range from small to medium at higher variance. Glomeruli of OR256-17 are exceedingly large, varying widely from medium to very large. Differences in glomerular size are highly significant with *p* < 0.0001 from OR256-17 to OR37A and OR18-2, respectively. Different Hashtag connotations (#,##) mark statistical groups significantly different from each other. **(C)** Positions of the glomeruli for OR37A, OR18-2 and OR256-17 on the anteroposterior and dorsoventral axes of the olfactory bulb (OB) are shown schematically from a sagittal view, representing the lateral part of the glomerular map. Glomeruli for OR37A are located at an anteroventral position, whereas dorsal OR18-2 glomeruli can be found in the middle segment of the OB and lateral glomeruli of OR256-17 reside in the posterior part of the OB. **(D)** Scatter plot depiction of the glomerular surface in relation to the number of DAPI stained nuclei for individual glomeruli of all three receptor types, the latter represented by different color. Glomeruli of OR37A are represented by green dots, those of OR18-2 by blue dots and OR256-17 by orange dots. The number of surrounding DAPI stained nuclei correlates with glomerulus size in a highly significant manner for all tested OR-specific glomerulus types (*p* < 0.0001).

**Figure 2:**
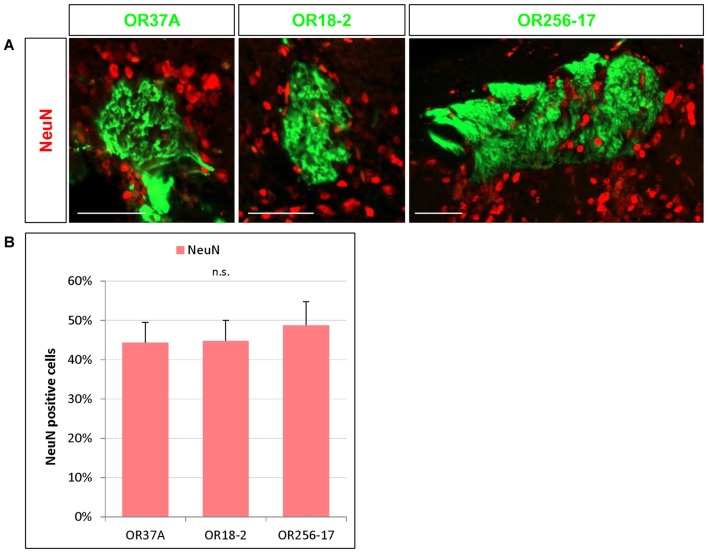
NeuN expressing cells in the periglomerular (PG) region. **(A)** Images of an OR37A, OR18-2 or OR256-17 glomerulus (green), respectively, with surrounding NeuN immunoreactive cells (red). Scale bar: 50 μm. **(B)** Quantification of NeuN positive cells in relation to the total of DAPI stained nuclei reveals a similar fraction of about 45% for all three glomerulus types.

**Figure 3:**
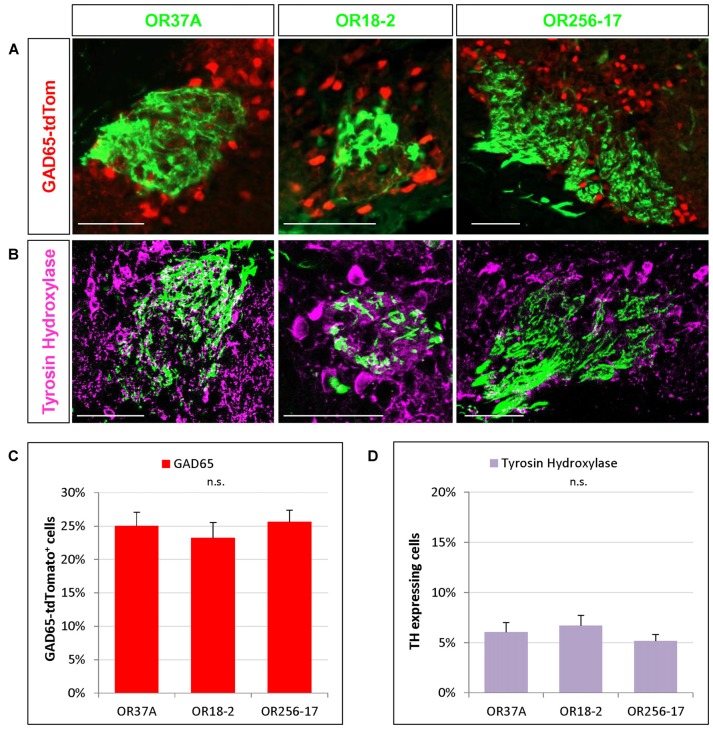
Cells expressing transmitter specific enzymes in the PG region. **(A)** Images of an OR37A, OR18-2 or OR256-17 glomerulus (green) respectively with surrounding transgenically labeled GAD65-tdTomato positive cells are shown (red). Scale bar: 50 μm. **(B)** Representative confocal single plane images of an OR37A, OR18-2 or OR256-17 glomerulus (green), respectively, with surrounding tyrosine hydroxylase (TH) immunoreactive neurons (purple). Scale bar: 50 μm. **(C)** Quantification of GAD65-tdTomato positive cells in relation to the total of DAPI stained nuclei yield percentages of about 25% for all three glomerulus types. **(D)** Quantification of TH positive cells in relation to the total of DAPI stained nuclei reveals similar fractions of about 6% for all three glomerulus types.

### Glomerular Surface Measurements

Glomerular surface measurements were conducted using ImageJ by measuring the outline of the respective glomerulus on each section throughout the structure. Single values were subsequently summed up and multiplied by 12 μm section thickness to receive a measure of the complete surface, as similarly described previously by another group (Bressel et al., 2016).

### Statistics

Statistical analyses were performed in SAS 9.4 software. Generalized linear mixed models with fixed effects for OR types and random effects for animal and glomerulus were applied. Boxplot representation of glomerular surface, shown in Figure 1B, was designed in SPSS 23 (IBM). Data for glomerular surface is shown in median and variance to match the boxplot representation. Any other data is shown as mean ± SD. *n* refers to the number of glomeruli analyzed in the regarding experiment. For detailed information on number of deployed animals for each experimental group, see Table 2. As glomeruli in reporter strains for OR37A do not show structural differences except for the reporter protein (Strotmann et al., 2000), data for OR37A-IRES-tau-GFP and OR37A-IRES-tau-lacZ were pooled for analyses. In no experiment any sex difference became apparent, so numbers were pooled for both sexes.

**Table 2:** Number of analyzed individuals.

Experiment	Mouse strain	# of individuals	# of glomeruli
*Glomerulus size and correlation*	OR37A-IRES-tau-GFP	6	8
	OR37A-IRES-tau-lacZ	4	5
	OR18-2-IRES-tau-GFP-tau-lacZ	6	21
	OR256-17-IRES-tau-GFP	8	19
*NeuN immunhistochemistry*	OR37A-IRES-tau-lacZ	3	9
	OR18-2-IRES-tau-GFP-tau-lacZ	4	18
	OR256-17-IRES-tau-GFP	4	12
*GAD65 analyses*	OR37A-IRES-tau-lacZ	4	10
	OR18-2-IRES-tau-GFP-tau-lacZ	4	5
	OR256-17-IRES-tau-GFP	6	22
*TH immunhistochemistry*	OR37A-IRES-tau-lacZ	5	16
	OR18-2-IRES-tau-GFP-tau-lacZ	4	12
	OR256-17-IRES-tau-GFP	4	9
*Parvalbumin immunhistochemistry*	OR37A-IRES-tau-GFP	4	9
	OR18-2-IRES-tau-GFP-tau-lacZ	4	25
	OR256-17-IRES-tau-GFP	3	18
*Calretinin immunhistochemistry*	OR37A-IRES-tau-GFP	4	6
	OR18-2-IRES-tau-GFP-tau-lacZ	4	21
	OR256-17-IRES-tau-GFP	4	16
*Calbindin immunhistochemistry*	OR37A-IRES-tau-GFP	3	6
	OR37A-IRES-tau-lacZ	1	3
	OR18-2-IRES-tau-GFP-tau-lacZ	4	17
	OR256-17-IRES-tau-GFP	3	11

*Number of glomeruli, in this study referred to as the number of *n*, and the corresponding number of individuals deployed in the regarding experiment is shown. The number of mice is shown per mouse strain, in which a specific glomerulus is labeled*.

## Results

### Comparison of Glomerulus Size

In this study we have analyzed the size of glomeruli for a member of the OR37 subsystem in comparison to glomeruli of class I and class II receptors, respectively. The glomeruli for each of these odorant receptors are located in very different, stereotyped positions along the rostrocaudal axis in the OB, as indicated in Figure 1C (see also Conzelmann et al., 2000; Strotmann et al., 2000; Zapiec and Mombaerts, 2015). Thereby, the selected glomerulus types not only represent different classes of odorant receptors but also different topological regions of the bulb. To visualize OR specific glomeruli, we used transgenic mouse lines in which GFP, lacZ or both are coexpressed with the corresponding odorant receptor (see also “Materials and Methods” section). In tissue sections derived from these mice, the respective glomeruli could be easily identified, as demonstrated in Figure 1A. In the course of analyzing such tissue sections, it became apparent that the glomeruli for OR37A, OR18-2 and OR256-17, respectively, significantly differed in size, as can be noted in Figure 1A. As a measure to evaluate diverging glomerulus sizes, we have determined the surface area of individual glomeruli. Similar to a procedure previously described by Bressel et al. (2016), the surface area was determined by measuring the glomerular outline on complete series of coronal sections multiplied by the thickness of the tissue sections. Using this approach, the surface dimension of 13 OR37A glomeruli was determined, resulting in a median value of 39.5*10^3^ μm^2^. Individual OR37A glomeruli showed a striking similarity in size, which is represented by the very low variance of 5.8*10^9^ μm^2^. The size of OR37A glomeruli is hence remarkably constant in view of glomeruli for other OR types. For OR18-2, the surface of 21 glomeruli was analyzed; the obtained sizes varied between small and medium with a median value of 25.3*10^3^ μm^2^. The variation in size was clearly higher than for OR37A; a variance of 19.4*10^9^ μm^2^ was determined. For OR256-17, relatively large glomeruli seem to be characteristic; a previous observation by Zapiec and Mombaerts (2015) was confirmed in this study. A rather high median value of 79.1*10^3^ μm^2^ was determined for OR256-17 glomeruli (*n* = 19). However, the sizes of 256-17 glomeruli varied considerably, ranging from medium to very large, with a variance of 127*10^9^ μm^2^. Examples of each, a large and a small version of a OR256-17 glomerulus are shown in Figure 1A. These results prove notable differences in the size of the glomeruli, which are highly significant with *p* < 0.0001. In Figure 1B, the corresponding data are presented in a boxplot depiction, which allows to appreciate the different degree of scattering of individual values for the size of the different glomerulus types. We hypothesized that the number of cells surrounding a respective glomerulus in the PG region might correlate with the size of the corresponding glomerulus. Therefore, we have determined the number of cells identified by DAPI staining in the immediate vicinity of individual glomeruli and correlated the obtained values to the respective glomerular surface. The results are depicted in Figure 1D and again emphasize the remarkably constant size of OR37A glomeruli. Concerning the number of cells relative to glomerulus surface, a very strong correlation for all tested OR specific glomerulus types with *p* < 0.0001 emerged from the analyses of 53 glomeruli. Hence, the number of cells stained by DAPI was considered a useful tool to acknowledge variations in glomerular dimension and was therefore deployed in subsequent analyses to relate cell counts to glomerulus size.

### NeuN Expressing Cells in the Periglomerular Region

Nuclear staining by DAPI labels any eukaryotic cell regardless of the subtype. Olfactory glomeruli however are also surrounded by a variety of glia cells (Valverde and Lopez-Mascaraque, 1991). To get an approximation how many of the DAPI stained cells in the close vicinity of the examined glomeruli may be neurons by immunhistochemical experiments using an antibody against the well-established neuronal marker NeuN (Mullen et al., 1992). Immunostaining for NeuN visualized numerous NeuN positive cells around each glomerulus, as is demonstrated in Figure 2A. The results of a quantification of the cells labeled for NeuN is documented in Figure 2B. The data indicate that 44.33 ± 5.19% of the DAPI stained nuclei surrounding OR37A glomeruli were NeuN positive (*n* = 9). A similar proportion (44.81 ± 5.22%) was obtained for OR18-2 glomeruli (*n* = 18) and a slightly higher ratio of 48.78 ± 5.97% was found for glomeruli of OR256-17 (*n* = 12). These data suggest that about half of DAPI stained cells in the PG region of the analyzed individual glomeruli are NeuN positive and that the proportion seems to be quite similar for all three odorant receptor specific glomerulus types.

### Cells Expressing Transmitter Specific Enzymes in the Periglomerular Region

In previous studies analyzing the glomerular layer of the bulb, it was reported that the majority of neurons in the PG region appear to be GABAergic (Panzanelli et al., 2007; Parrish-Aungst et al., 2007). However, any regional distinctions regarding different zones of the bulb or distinct glomeruli have not been made. Therefore, in this study we have determined the proportion of GABAergic neurons around individual glomeruli which are typical for distinct OR types and located in different zones of the bulb. To visualize GABAergic neurons, we used a transgenic mouse line expressing the red fluorescent protein tdTomato under the control of the promoter for the GAD65 gene (Besser et al., 2015). GAD65 is one of two isoforms of glutamate decarboxylase, a key enzyme for the synthesis of GABA and is widely accepted as a marker for GABAergic neurons (Erlander et al., 1991; Kiyokage et al., 2010). The results of experiments visualizing labeled cells around individual glomeruli are demonstrated in Figure 3A. Determining the number of GAD65 positive cells relative to the number of DAPI stained nuclei in the PG region revealed that a portion of 25.01 ± 2.07% of the cells around glomeruli of OR37A was GAD65 positive (*n* = 10). For OR18-2 glomeruli, the proportion of GAD65 positive cells was slightly lower at 23.22 ± 2.35% (*n* = 5) and for OR256-17 glomeruli, a proportion of 25.63 ± 1.76% GAD65 positive cells was determined (*n* = 22). The results are depicted in Figure 3C. These data indicate that the proportion of GAD65 positive cells is quite similar for all three examined glomerulus types; i.e., about a quarter of all cells visualized by DAPI in the immediate surrounding of the respective glomeruli. Furthermore, a subset of neurons in the OB has been shown to be dopaminergic (Parrish-Aungst et al., 2007; Kiyokage et al., 2010; Borisovska et al., 2013). Dopaminergic neurons can be visualized by an antibody against TH, the rate-limiting enzyme for the synthesis of dopamine (McLean and Shipley, 1988; Parrish-Aungst et al., 2007; Pignatelli and Belluzzi, 2017). We adapted this approach to identify TH expressing cells in the PG region of distinct glomeruli. In immunostained sections several TH positive cells in the vicinity of individual glomeruli became visible (Figure 3B). Evaluating the number of TH labeled cells relative to the number of DAPI stained nuclei resulted in a portion of 6.05 ± 0.95% TH positive cells around OR37A glomeruli (*n* = 16), a portion of 6.7 ± 1.02% for glomeruli of OR18-2 (*n* = 12) and a fraction of 5.18 ± 0.64% for OR256-17 glomeruli (*n* = 9; Figure 3D). These results indicate that TH immunoreactive cells account for about 6% of the cells in the PG region; obviously quite similar for all three odorant receptor specific glomerulus types analyzed.

### Cells Expressing Calcium-Binding Proteins in the Periglomerular Region

**Figure 4:**
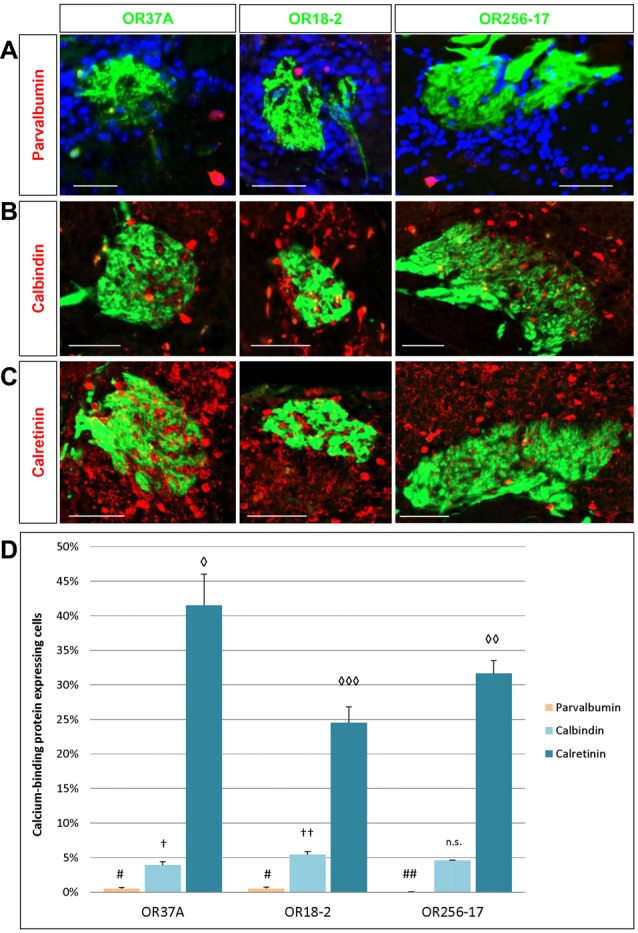
Cells expressing calcium-binding proteins in the PG region. **(A)** Images of an OR37A, OR18-2 or OR256-17 glomerulus (green), respectively, with parvalbumin (PV) immunoreactive cells (red) and DAPI staining (blue). Scale bar: 50 μm. **(B)** Images of an OR37A, OR18-2 or OR256-17 glomerulus, respectively, with surrounding calbindin (CB) immunoreactive cells (red). Scale bar: 50 μm. **(C)** Images of an OR37A, OR18-2 or OR256-17 glomerulus (green), respectively, with surrounding calretinin (CR) immunoreactive cells (red). Scale bar: 50 μm. **(D)** Quantification of the portion of calcium-binding protein expressing cells. PV positive cells in relation to the total of DAPI stained nuclei result in particularly low numbers and are here, other than in the results section, shown in percentages for a better comparison with the data for CB and CR. Values for PV+ cells are represented by yellow bars. OR37A and OR18-2 harbor a significantly higher number of PV+ neurons in their vicinity than OR256-17 (*p* = 0.0075 and *p* = 0.0068 respectively). The quantification of CB positive cells in relation to the total of DAPI stained nuclei shows a significantly diverging proportion for every receptor-specific glomerulus type, with the lowest percentage around OR37A glomeruli and the highest around OR18-2 glomeruli (OR37A to OR18-2: *p* = 0.032; OR256-17 to OR18-2: *p* = 0.089). Percentages for CB expressing cells are represented by light blue bars. Quantification of CR positive cells reveals an inversed distribution pattern, albeit at considerably higher numbers than the first, representing the most numerous subpopulation as indicated by the dark blue bars. CR positive cells exhibit the significantly highest ratio around OR37A glomeruli and the lowest around OR18-2 glomeruli (OR37A to OR18-2: *p* = 0.002; OR37A to OR256-17: *p* = 0.048; OR256-17 to OR18-2: *p* = 0.041). Symbol connotations (#,†,◊) mark statistical groups individually per dataset, varying number of the same symbol indicating a statistical significant difference between two groups.

The expression of distinct calcium-binding proteins has previously been used to define certain subtypes of neurons in the glomerular layer of the OB (Parrish-Aungst et al., 2007; Nagayama et al., 2014). Here, we have analyzed the expression pattern of three subtypes of calcium-binding proteins, namely parvalbumin (PV), calretinin (CR) and calbindin (CB), in the PG region of OR specific glomeruli. Cells expressing the respective proteins were visualized by immunostainings using specific antibodies. The resulting staining patterns are depicted in Figures 4A–C. Towards a better comprehension of the individual populations, the number of labeled cells in relation to DAPI stained cells was determined. The results of a quantification for all three subtypes are shown in Figure 4D. Although PV expressing cells are supposed to reside predominantly in the external plexiform layer where they form spatially widespread connection patterns (Kosaka and Kosaka, 2008; Miyamichi et al., 2013), a small population of PV expressing cells has also been observed in the glomerular layer. This cell type has rarely been analyzed due to the low numbers and usually relatively weak labeling intensity (Crespo et al., 1997; Baltanás et al., 2007; Parrish-Aungst et al., 2007). Yet, application of a specific antibody against PV resulted in a substantial labeling of single PV positive cells in the PG area of all three glomerulus types, as can be seen in Figure 4A. A quantification of the labeled cells was performed; the results are depicted in Figure 4D. As expected, the numbers were very low. Therefore, the proportion of PV expressing cells here is given as absolute counts per 1000 DAPI stained cells. In the close vicinity of OR37A glomeruli, 4.97 ± 2.16 PV positive cells were detected per 1000 DAPI stained cells (*n* = 9) and 5.3 ± 2.19 for OR18-2 glomeruli (*n* = 25). Although these numbers might seem to be quite low, they nevertheless are significantly higher (*p* = 0.0075 and *p* = 0.0068) than that for OR256-17 with a mean of 0.38 ± 0.22 (*n* = 18). Thus, the proportion of PV expressing cells in the PG region seems to vary among the different glomerulus types. Another subpopulation of neurons in the glomerular layer is characterized by the expression of CB (Kosaka and Kosaka, 2010). In an immunhistochemical approach using a specific antibody, CB expressing cells were visualized in the vicinity of the examined glomeruli (Figure 4B). Quantitative assessment of labeled cells yielded a strikingly low value for glomeruli of OR37A, with a mean of 3.92 ± 0.5% CB positive cells (*n* = 9). For OR18-2 glomeruli, a percentage of 5.45 ± 0.41% was obtained with *p* = 0.032 (*n* = 17), whereas the percentage for OR256-17 glomeruli ranges at a medium level of 4.61 ± 0.03% (*n* = 11). These results are depicted in Figure 4D and suggest that the CB expressing cells show distinct distribution patterns for OR specific glomerulus types. The CR expressing cells turned out to be the most numerous among the calcium-binding protein expressing cell populations examined in this study. Immunostainings for CR labeled a substantial number of cells in the immediate surrounding of OR37A, OR18-2 and OR256-17 glomeruli (Figure 4C). Assessing the number of CR expressing cells revealed that the highest proportion of CR expressing cells was found around OR37A glomeruli with a mean of 41.52 ± 4.52% (*n* = 6; Figure 4D). This value was significantly higher than that for OR256-17 (*p* = 0.048) and OR18-2 (*p* = 0.002), respectively. OR18-2 glomeruli featured only a quite low portion of CR positive cells of 24.52 ± 2.3% (*n* = 21). The proportion of positive cells around OR256-27 glomeruli was determined as 31.65 ± 1.87% (*n* = 16). These results indicate that the fraction of CR expressing cells varied significantly among the OR specific glomerulus types. Together, the findings of this study indicate that the proportion of transmitter specific neurons is quite consistent around specific glomeruli, whereas the cell populations characterized by distinct calcium binding proteins varied significantly for distinct OR specific glomeruli.

## Discussion

Based on extensive recent studies it has been proposed that individual olfactory glomeruli may possess a complex network of PG cells which operate as specific microcircuitries adapted to process distinct odor representations (Wachowiak and Shipley, 2006; Linster and Cleland, 2009). In the present study, we have analyzed structural glomerular features and PG cell types of an OR37 glomerulus in comparison to those of two different odorant receptors, which represent class I and class II odorant receptors, respectively. The three receptor types differ from one another in several aspects, including the nature of their ligands, their tuning profiles to ligands and their topographic expression patterns. OR37A, as other members of the OR37 family, responds primarily to a distinct ligand, namely pentadecanal (Bautze et al., 2012); this long chain fatty aldehyde is considered to play a role in social communication (Bautze et al., 2014; Klein et al., 2015). The class I odorant receptor OR18-2 has been shown to detect metabolic products, such as short chain fatty acids or lactate (Pluznick et al., 2013; Fleischer et al., 2015). The class II odorant receptor OR256-27 has been demonstrated to be broadly tuned and responds to a large variety of odorants, including environmental cues (Tazir et al., 2016). Furthermore, the glomeruli of these three odorant receptor types are located in very different positions within the OB, thus representing topographically distinct areas of the bulb. The size of the three glomerulus types differed significantly; rather small glomeruli were found for OR18-2, medium sized glomeruli for OR37A and very large glomeruli for OR256-17. Comparing the size of glomeruli in individual mice revealed notable differences regarding the variability of the glomerulus size for the three types; the glomeruli for OR37A stood out for remarkably low variations in their size. Since the size of a given glomerulus seems to correlate linearly to the number of sensory neurons in the olfactory epithelium which express the corresponding odorant receptor (Bressel et al., 2016), the exceptionally constant size of the OR37A glomeruli in individual mice suggests a relatively constant number of neurons in the olfactory epithelium expressing the OR37A receptor type. This view is in line with a recent observation, which indicated that the number of neurons expressing OR37C was very similar among individual mice (Bressel et al., 2016). As a consequence, it seems that for neurons expressing a receptor type of the OR37 family, not only the spatial distribution in a small patch of the olfactory epithelium (Strotmann et al., 1999), but also the number of cells and consequently the glomerulus size seems to be strictly regulated. This appears to be another unique feature of the OR37 subsystem, since the glomeruli for OR18-2 and OR256-17 showed a significantly higher degree of variation in their size. We deduced that the number of cells surrounding the regarding glomerulus, i.e., the number of interneurons involved in neuronal processing, might be correlated with the size of a glomerulus. The analyses confirmed that notion, demonstrating that the number of cells in the close vicinity of a given glomerulus strongly correlated with the glomerular size. Based on this clear correlation, the number of surrounding cells may be a useful indicator for glomerular size in further studies. Since olfactory glomeruli are surrounded by distinctive glia formations (Valverde and Lopez-Mascaraque, 1991), a substantial proportion of the DAPI stained cells in the close vicinity of the glomeruli are probably glia cells. Therefore, we sought to determine the proportion of neurons in the vicinity of the specific glomeruli by immunostainings against the neuronal marker NeuN. Although possibly not all neurons might be visualized by NeuN (Panzanelli et al., 2007), we considered NeuN staining a useful approach to get a landmark for the proportion of neuronal cells around individual glomeruli in comparison to one another. Based on our results, at least half of all DAPI stained cells can be considered neurons. The majority of neurons in the PG region appear to be GABAergic (Panzanelli et al., 2007; Parrish-Aungst et al., 2007). As a measure for GABAergic neurons in the surrounding of the glomeruli, the number of GAD65 positive cells was determined resulting in a range of about 25% of all cells in the PG region for all glomerulus types. Based on the number of NeuN positive cells, this data suggests that merely half of the neurons in the PG region are GABAergic. The total number of GABAergic neurons may be higher though, since a substantial subset of PG neurons is supposed to express the GAD67 isoform (Panzanelli et al., 2007; Kiyokage et al., 2010). Nevertheless, the analyses of GAD65 positive cells can be considered as a useful approach to compare the relative proportions of GABAergic neurons for receptor specific glomeruli. For GAD65 neurons, it has been proposed that they may be involved in gain control mechanisms in individual glomeruli (Kiyokage et al., 2010). Thus, the results of this study might imply that a similar gain control capacity may exist in different glomerulus types. Dopaminergic neurons in the glomerular layer of the OB are supposed to span over the range of several glomeruli, making multiglomerular contacts (Kiyokage et al., 2010; Pignatelli and Belluzzi, 2017). For all three glomerulus types, the relative population of tyrosine hydroxylase positive cells was similar in the range of about 6% TH positive cells in the PG region. This result implies that the population of dopaminergic neurons in the PG area is consistent for the different glomerulus types. The expression of distinct calcium-binding proteins has been widely used as a criterion for categorizing the various cellular subtypes in the glomerular layer (Baltanás et al., 2007; Parrish-Aungst et al., 2007). Here we evaluated the relative proportion in the PG region for cell subpopulations expressing either PV, CR or CB, respectively. Although PV expressing neurons in the OB are typically most prominent in the external plexiform layer, individual PV positive cells have also been observed in the glomerular layer (Crespo et al., 1997; Baltanás et al., 2007; Kato et al., 2013). In the present study, a significant number of PV positive PG cells was found for glomeruli of OR37A and OR18-2, whereas for OR256-17 rarely any PV positive cell could be detected. This finding suggests that the small subset of PV expressing cells in the glomerular vicinity may play a specific role for certain glomerulus types. Thus, exploring the functional properties of PV positive PG cells might contribute to elucidate neuronal networks of odorant receptor specific glomeruli. The number of CB positive cells in the periphery of individual glomeruli was found to vary significantly, with a strikingly low value for OR37A glomeruli and the highest value for OR18-2 glomeruli. CB positive cells are supposed to be involved in gain control processes of individual glomeruli (Kosaka and Kosaka, 2010; Nagayama et al., 2014); thus, this cell type may play a distinct role in this neuronal process as their proportion differed significantly for the different glomerulus types. CR positive cells represented the most numerous population of calcium binding protein defined subtypes for all three glomerulus types. The significantly highest proportion of over 40% of all cells in the PG region was found for OR37A. CR expressing cells are proposed to be involved in regulating the signal-to-noise ratio in activated glomeruli (Fogli Iseppe et al., 2016). A high population of CR positive cells might thus lead to a pronounced depression of unspecific signals, thus enhancing the discrimination capacity of a glomerulus. Intriguingly, the high population of CR positive cells in the vicinity of OR37A glomeruli is in accordance with the notion that OR37A is activated only by specific compounds (Bautze et al., 2012). In summary, the proportion of the three subpopulations of calcium-binding protein expressing cells in the PG region of odorant receptor specific glomeruli differed significantly. Interestingly, PG cells defined by the expression of either CR or CB are considered type II PG cells making no direct synaptic contacts with olfactory sensory axons, whereas TH expressing PG cells are classified as type I which is additionally directly driven by olfactory nerve terminals (Kosaka and Kosaka, 2007; Shao et al., 2009). Given the fact that only calcium-binding protein defined cell types differed in their relative numbers around certain glomeruli, it seems that the specific repertoire of those subtypes, which presumably represent type II PG cells, might contribute to the formation of a glomerulus specific microcircuitry of interneurons. Collectively, the results of the present study indicate that individual odorant receptor specific glomeruli are dissimilar in size and show significant differences concerning the variability of their size. In addition, the different glomerulus types are surrounded by a quite distinct PG network contributing to the processing of distinct odor qualities. OR37A glomeruli stood out by a remarkably stable size in different individuals, which may reflect a relatively fixed number of sensory neurons expressing OR37A in the olfactory epithelium. Furthermore, glomeruli for OR37A were characterized by a distinct repertoire of calcium binding protein defined cell types in the PG region. These findings further support the view that the OR37 subsystem represents a unique part of the main olfactory system.

## Author Contributions

A-MM, HB and JS conceived and designed the experiments; A-MM performed the experiments, analyzed the data and prepared the figures; A-MM and HB wrote the manuscript. All authors approved the final version of the manuscript.

## Conflict of Interest Statement

The authors declare that the research was conducted in the absence of any commercial or financial relationships that could be construed as a potential conflict of interest.
